# The Eμ-Ret mouse is a novel model of hyperdiploid B-cell acute lymphoblastic leukemia

**DOI:** 10.1038/s41375-024-02221-x

**Published:** 2024-03-22

**Authors:** Ali Farrokhi, Tanmaya Atre, Jenna Rever, Mario Fidanza, Wendy Duey, Samuel Salitra, Junia Myung, Meiyun Guo, Sumin Jo, Anuli Uzozie, Fatemeh Baharvand, Nina Rolf, Franziska Auer, Julia Hauer, Stephan A. Grupp, Patrice Eydoux, Philipp F. Lange, Alix E. Seif, Christopher A. Maxwell, Gregor S. D. Reid

**Affiliations:** 1 Michael Cuccione Childhood Cancer Research Program, BC Children’s Hospital Research Institute, Vancouver, BC Canada; 2 Department of Pathology and Laboratory Medicine, University of British Columbia, Vancouver, BC Canada; 3 Department of Pediatrics, Children’s Cancer Research Center, Kinderklinik München Schwabing, School of Medicine, Technical University of Munich, Munich, Germany; 4 Abramson Family Cancer Research Institute, University of Pennsylvania, Philadelphia, PA USA; 5 Department of Pediatrics, University of British Columbia, Vancouver, BC Canada

**Keywords:** Cancer models, Preclinical research, Oncogenesis, Acute lymphocytic leukaemia

## Abstract

The presence of supernumerary chromosomes is the only abnormality shared by all patients diagnosed with high-hyperdiploid B cell acute lymphoblastic leukemia (HD-ALL). Despite being the most frequently diagnosed pediatric leukemia, the lack of clonal molecular lesions and complete absence of appropriate experimental models have impeded the elucidation of HD-ALL leukemogenesis. Here, we report that for 23 leukemia samples isolated from moribund Eμ-Ret mice, all were characterized by non-random chromosomal gains, involving combinations of trisomy 9, 12, 14, 15, and 17. With a median gain of three chromosomes, leukemia emerged after a prolonged latency from a preleukemic B cell precursor cell population displaying more diverse aneuploidy. Transition from preleukemia to overt disease in Eμ-Ret mice is associated with acquisition of heterogeneous genomic abnormalities affecting the expression of genes implicated in pediatric B-ALL. The development of abnormal centrosomes in parallel with aneuploidy renders both preleukemic and leukemic cells sensitive to inhibitors of centrosome clustering, enabling targeted in vivo depletion of leukemia-propagating cells. This study reveals the Eμ-Ret mouse to be a novel tool for investigating HD-ALL leukemogenesis, including supervision and selection of preleukemic aneuploid clones by the immune system and identification of vulnerabilities that could be targeted to prevent relapse.

## Introduction

Almost one third of children diagnosed with B cell acute lymphoblastic leukemia (B-ALL) present with the high-hyperdiploid sub-type (HD-ALL), making it the largest contributor to the unique incidence peak of B-ALL at 3–5 years of age [1, 2]. HD-ALL is characterized by non-random chromosomal gains, with combinations of chromosomes X, 4, 6, 10, 14, 17, 18, and 21, mostly in the form of trisomies, accounting for 75% of the changes [3, 4]. Chromosomal content is associated with prognosis and the pattern of gains and losses is increasingly used for risk stratification [5, 6]. In contrast to other aneuploid malignancies, the abnormal karyotype is relatively stable through HD-ALL progression. However, sub-clonal heterogeneity in chromosome number and the presence of markers of chromosomal instability (CIN) have been observed in patient samples, suggesting mitotic defects may contribute to clonal evolution during disease progression [7–11].

Current evidence supports that pediatric HD-ALL aneuploidy is seeded by a single aberrant mitotic event that in most cases occurs in utero [12–15]. The observation that aneuploidy usually precedes the acquisition of potential driver mutations indicates that the gain of chromosomes is likely the initiating event in HD-ALL leukemogenesis [4, 16]. Studies of twins revealed that leukemia onset is associated with the independent acquisition of a second genetic event within a shared aneuploid preleukemic cell population [15, 17, 18]. The contribution of an expanded preleukemic population to HD-ALL progression is also detected later in disease: relapse clones are characterized by an increased number of structural chromosomal abnormalities (including sub-chromosomal duplications and microdeletions) and uniparental isodisomies compared to those at diagnosis [19, 20]. In the majority of patients, the pattern of genomic variants indicates that the dominant clones present at diagnosis and relapse evolved independently from a common ancestral clone [19].

Aneuploidy is the only genetic anomaly common to all HD-ALL patients [3]. Mutation burden is low in HD-ALL, but variants affecting RAS-signaling and histone-modifier genes have been identified as potential contributors to leukemia progression [4, 16, 19, 21–24]. However, these mutations are mostly sub-clonal and are absent in almost half of all cases. Due to the lack of experimental models, investigation of HD-ALL leukemogenesis has been limited to the study of fully transformed leukemia cells and retrospective detection of specific characteristics (e.g. particular trisomies or IgH rearrangements) during the preleukemic phase of disease. As a result, in comparison to other ALL subtypes with defined molecular drivers, the leukemogenesis process of HD-ALL is less well understood and specific, broadly applicable targeting strategies remain to be identified [3, 25]. Here, we report that the Eμ-Ret transgenic mouse is a model of hyperdiploid B cell precursor (BCP) ALL with notable similarities to clinical disease [26–28]. Further, we demonstrate that both preleukemic and leukemic BCP cells are sensitive to the inhibition of pathways involved in the handling of centrosome amplification (CA), revealing a potential strategy to deplete the residual clones that drive HD-ALL relapse.

## Materials and methods

### Mice

BALB/c and NOD-scid/IL2Rγ null (NSG) mice were obtained from Jackson Laboratory (Bar Harbor, ME). Eμ-Ret mice were maintained as transgene hemizygotes on a BALB/c background by in-house breeding [27]. Both male and female mice were used in all experiments. Overt hematologic disease was defined as a white blood cell count (WBC) of >20,000/μl or the presence of palpable lymph nodes. For leukemia-initiating studies, sorted preleukemia cells (1–3 × 10^5^) were injected intravenously into NSG mice. All longitudinal disease progression monitoring was achieved by flow cytometric analysis of peripheral blood. Mice were euthanized prior to or at detection of leukemic end-points and evaluated by flow cytometry for preleukemic or leukemic BCP cell burden in the indicated organs by flow cytometry. Where indicated, adult (5–7 weeks old) Eμ-Ret mice were treated with four 30 mg/kg doses of AZ82 or vehicle (5% DMSO in saline) by intraperitoneal (i.p.) injection over an eight-day period and then euthanized 24–48 h after the last injection. Bone marrow and spleen were analyzed by flow cytometry for the presence of BCP cell subsets. Cell counts were quantified using CountBright beads (ThermoFisher, Waltham, MA). All experiments were conducted in accordance with a University of British Columbia Animal Care Committee-approved protocol (A19-0197).

### Flow cytometry

Splenocytes and bone marrow cells were stained with combinations of the following fluorochrome-conjugated antibodies for B220 (RA3-6B2), BP-1 (6C3), CD24 (M1/69), IgM (RMM-1), CD117 (ACK2), CD25 (PC61), and CD19 (6D5) (BioLegend, San Diego, CA), and CD43 (S7) (BD Biosciences, San Jose, CA). 7AAD was included for viability assessment. Flow data were acquired on Accuri C6, LSRII, or Fortessa cytometers (BD Biosciences) and analyzed using FlowJo software v10.8 (BD Bioscience) using established gating strategies (Supplemental Fig. S1). Flow cytometric sorting of preleukemic BCP cells, based on their characteristic B220^int^/CD43^int^/BP-1^hi^ phenotype, routinely achieved purity >90% on a MoFlo Astrios EQ cell sorter (Beckman Coulter Inc., Brea, CA, USA).

### In vitro studies

In vitro experiments were performed with preleukemic cells enriched from bone marrow (BM) or spleens of healthy Eμ-Ret mice. Leukemic cells were isolated from the spleens of moribund Eμ-Ret mice; in most cases, leukemia cells with the BCP phenotype constituted >80% of the splenic cells and no enrichment was performed. BM stromal cells were harvested from the femur and tibia of BALB/c mice by aspiration with saline solution. Cells were cultured in complete Dulbecco’s Modified Eagle Medium (DMEM, Gibco, Waltham, MA) supplemented with 20% FBS, 2mM l-glutamine, 20 U/mL penicillin, 20 μg/mL streptomycin, 20 mM HEPES, 1× NEAA, and 91.5 μM 2ME, and 250 pg/mL IL-7 (Sigma, St. Louis, MO). For proliferation assays, 5 × 10^4^ leukemic or sorted preleukemic cells were cultured with 5 × 10^5^ BM stromal cells in RPMI supplemented with or without 10 μg/ml anti-IL-7Rα antibody (Clone A734, BioXcell, Lebanon, NH) for 72 h and the viable BCP cells counted. For drug sensitivity assays, the protocol described by Frismantas et al. was adapted to a 96-well format, as described previously [29, 30]. BM stromal cells were seeded at 5000 per well in 200 μL of stromal cell media 24 h prior to seeding with 50,000 primary preleukemia, leukemia, or Eμ-Ret-derived B-ALL cell line 289 cells. Cell cultures were incubated with a serial dilution of inhibitor drugs AZ82 and CW069 (MedChemExpress, Monmouth Junction, NJ), and Griseofulvin, BI2536, or MLN8237 (Selleck Chemicals, Houston, TX) at 37 °C in a 5% (v/v) CO_2_ incubator for 48 h. Cell viability was quantified with CyQUANT Direct Cell Proliferation Assay (ThermoFisher Scientific, Waltham, MA).

### RNA sequencing

RNA was isolated from 4 preleukemic and 3 leukemic Eμ-Ret BCP cell samples. RNA sequencing (RNA-seq) was performed at the University of British Columbia Biomedical Research Centre. Bulk RNA-Seq data were analyzed using the “DESeq2” R package [31, 32]. Unqualified genes were filtered out (based on a summation through all samples of less than 10 counts) prior to running the “DESeq” function. Shrunken estimates of log2 fold changes were generated using the “lfcShrink” function with the “ashr” method. Genes with FDR < 0.05 were identified as differentially expressed genes (DEGs). The “rlog” function was used to transform the count data to log2 scale and normalize data. Principal component analysis (PCA) was then performed to reduce the dimensions of the data to cluster the samples visually. Gene Set Enrichment Analysis (GSEA) was performed to investigate the significantly impacted biological pathways using the “clusterProfiler” and “meshes” R packages [33, 34]. The “gseMeSH” function was used for GSEA with MeSH (Medical Subject Headings) terms from the “Phenomena and Processes” category. The analysis was performed for significantly upregulated genes (log2 fold change >0) and down-regulated genes (log2 fold change < 0) separately. The enriched terms were visualized using the “enrichplot” R package.

### Whole exome sequencing

Whole-exome sequencing (WES) was carried out as previously described [35]. DNA extracted from the tail of the respective mice was used as a germline control for somatic analyses. Based on our previous experience [35], the sensitivity to distinguish between tumor and germline control is not sufficient for somatic variant calls with less than 9% difference in variant allele frequency (VAF). Therefore, such variants were removed from the final results. See Supplemental Material for details.

### Genomic analysis

Genomic DNA from 18 Eμ-Ret B-ALL and 6 transgene-negative BALB/c control B cell samples was analyzed by chromosome microarray (CMA) at the Fox Chase Cancer Center Genomics facility. CEL file output from the Affymetrix Mouse Diversity Genotyping Array was analyzed using the Golden Helix SNP and Variation Suite (SVS) analysis software (Bozeman, MT). Intensity data was interpreted directly from the raw file source to call and characterize copy number variants (CNV) in each sample, and resulting visualizations were generated using the standard workflows in the Golden Helix software package. For optical genome mapping (OGM), high molecular weight DNA from 3 additional Eμ-Ret leukemia samples was analyzed on the Saphyr platform by Bionano Genomics (San Diego, CA), using their rare variant analysis workflow. Data for structural variation detection was processed using Bionano Access software.

### Karyotyping

Samples from mice were processed by direct-harvest methods to obtain metaphase chromosomes for analysis. Metaphase preparations were stained using Trypsin-Giemsa banding and a minimum of 3 and a maximum of 30 metaphases were analyzed per sample. Samples included 7 preleukemic and 2 leukemic samples, and 2 control samples of non-leukemic B cells isolated from healthy Eμ-Ret mice. See Supplemental Material for details. Multicolor Fluorescence In situ Hybridization (M-FISH) slides were prepared using the same fixed cell suspensions that had been used for G-banding analysis. Slides were pretreated with 0.01 N HCL/pepsin solution and washed with PBS, followed by sequential dehydration with 70%, 85%, and 100% ethanol. Hybridization with the 21XMouse Multicolor FISH probe (MetaSystems, Heidelberg, Germany) utilized the ThermoBrite automated denaturation and hybridization system. Following post-hybridization washes, slides were counterstained with DAPI. Metaphase cell capture and analysis was performed using the Metasystems M-FISH software.

### Proteomic analysis

Proteins were extracted from purified leukemic and preleukemia cells in 50 mM HEPES buffer, pH8.5 containing 1% SDS (Fisher BioReagents, Pittsburgh, USA) and protease inhibitor (Pierce, Waltham, MA). The lysate was incubated at 37 °C for 30 min followed by reduction and alkylation using 50 mM TCEP and CAA (Sigma), respectively. Protein concentration was estimated using BCA assay (Sigma). Prepared samples were processed for clean-up and trypsin digestion using SP3 through the addition of Sera-Mag Speed Beads (GE Life Sciences, Chicago, IL) (See Supplemental Material for details). The eluted peptides were separated using a 60 min active gradient from 0–34% Buffer B (0.1% FA in 95% acetonitrile) on a EasyNLC 1200 with a 50µPAC column (ThermoScientific) coupled to a Q Exactive HF orbitrap mass spectrometer. Data was acquired by Data-independent acquisition (See Supplemental Material for details). Statistical significance was determined using the Student’s *t*-test as implemented in Spectronaut. STRING (https://www.string-db.org) was used to determine proteins interacting with KIFC1. Proteins selected from STRING with evidence of direct experimentally-shown interaction were visualized in volcano plot using R with the Log2-transformed intensities and Log10 of the q-value of each protein on the x-axis and y-axis, respectively.

### Immunofluorescence evaluation of centrosome amplification

Cells were concentrated onto a slide using the Epridia Cytospin 4 centrifuge and fixed in methanol at −20 °C for 5 min. Cells were blocked in PBS with 0.2% Triton X-100 and 3% BSA for 1 h at room temperature. Antibodies were diluted in PBS with 0.2% Triton X-100 and 3% BSA. Primary antibodies were diluted and incubated with slides overnight at 4 °C. Cells were then washed three times in PBS. The slides were incubated with diluted secondary antibodies at room temperature for 1 h in the dark. Slides were washed three times in PBS and incubated with Hoechst stain for 15 min, then washed two times in PBS and coverslips were mounted with ProLong Gold Antifade reagent (Invitrogen, Waltham, MA). Immunofluorescence analysis was used to stain centrosomes (gamma-tubulin), and emanating microtubules (tubulin) in both interphase and mitotic cells. Additionally, different components of the centrosome were stained, including pericentrin (PCNT) and centrobin (CNTROB). Immunofluorescence was also used to identify mitotic cells (phospho-histone H3), as well as quantify KIFC1 abundance.

### Confocal microscopy and image acquisition

Fixed cells were imaged using the Fluoview software (Olympus, Tokyo, Japan) connected to the Olympus Fluoview FV10i confocal microscope. Image stacks of optical sections with a spacing of 0.5 μm through the cell volume were taken using a 60 × 1.2 NA oil objective. All images were taken at an image size of 1024 × 1024 pixels, a scan speed and quality (average) setting of Balance (x4), and a confocal aperture of ×1.5. ImageJ v1.46j (National Institute of Health, Bethesda, MD) was used to generate maximum intensity projections of the fluorescent channels. To identify centrosomes in interphase and mitotic cells, slides were stained with gamma-tubulin antibody and AlexaFluor 488, and centrosome area was measured by drawing (Freehand selections) a ROI around each centrosome identified in the gamma-tubulin channel (Measure, ImageJ). Additionally, for some experiments, centrosome area was measured by drawing a ROI around each centrosome identified in the merged image of the gamma-tubulin, pericentrin, and centrobin channels. To identify mitotic cells for cell cycle-specific confocal analysis, cells were stained with phospho-histone H3 (pH3) antibody conjugated to AlexaFluor 594. To determine fluorescence intensity, cells stained with KIFC1 antibody and AlexaFluor 488 were imaged at 50% sensitivity and 40% laser power for all replicates to allow for comparisons between images. For nucleus specific analysis, nuclear masks were generated (Make Binary, ImageJ) from cells stained with the nuclear dye Hoechst, and the resulting region of interest (Analyze Particles, ImageJ) was used to identify and measure the nuclear region of analysis.

### Statistical analysis

**Fig. 1:**
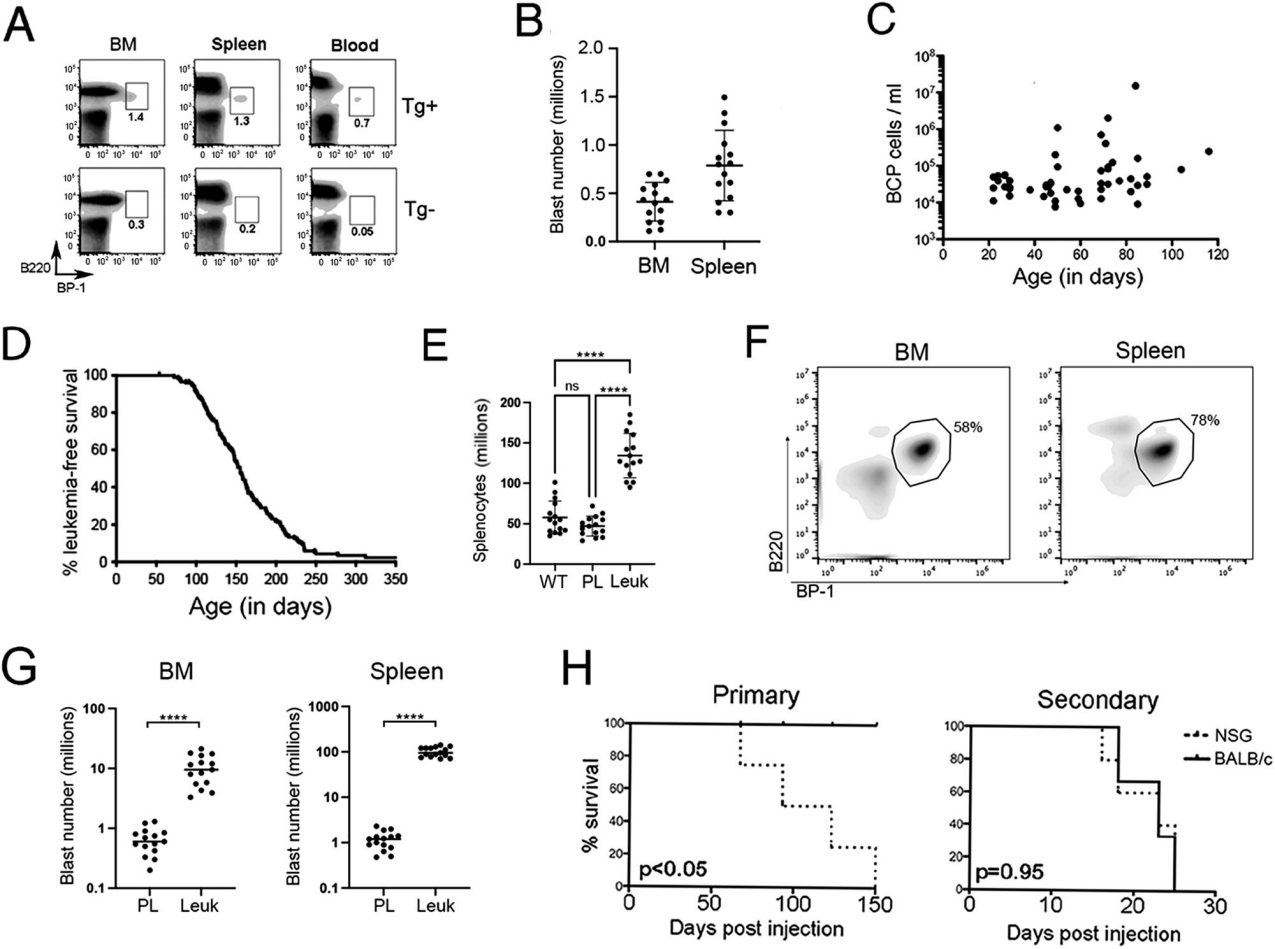
Eμ-Ret leukemia progression characteristics. **A** An expanded BCP population is detectable in bone marrow (BM), spleen, and peripheral blood of 4-week-old Eμ-Ret mice (Tg+), based on their characteristic cell surface phenotype, compared to their transgene-negative (Tg-) littermates. Numbers shown indicate BCP cells as % of total viable cells. **B** The size of the preleukemic cell population shows considerable variation between individual 4-week-old Eμ-Ret mice (*n* = 15). **C** When monitored over the first 120 days, no significant expansion of the abnormal BCP population is observed before 65 days of age, the earliest age of onset on leukemia in this model (*p* = 0.7412), or afterwards (*p* = 0.3016), using a linear mixed effects model. The concentration of preleukemic BCP cells in peripheral blood was measured for each Eμ-Ret mouse at a single time-point; no mouse contributed more than one data point (*n* = 45). **D** Leukemia progression kinetics in 182 Eμ-Ret mice housed under SPF-conditions in 2 separate facilities. Leukemia is >95% penetrant in Eμ-Ret mice on a BALB/c background within one year. **E** Leukemic Eμ-Ret mice present with significantly higher number of cells in their spleens compared to wild-type (WT) BALB/c and preleukemic Eμ-Ret controls **(**PL). **F** The cellularity of organs at time of leukemia onset is dominated by leukemic blasts with the characteristic abnormal BCP phenotype. **G** Numbers of blasts (identified by their B220^int^/CD43^int^/BP-1^hi^ phenotype) in bone marrow and spleen of leukemic mice are significantly higher than in healthy Eμ-Ret mice of similar age (*n* = 15 for both). **H** Survival of NSG and BALB/c recipients injected with 1–3 × 10^5^ preleukemia cells obtained from young Eμ-Ret mice (Primary, left panel) or with 10^5^ leukemic cells that developed in the primary NSG recipients (Secondary, right panel). Four NSG and 4 BALB/c mice received preleukemia cells and 5 NSG and 6 BALB/c mice received the resultant leukemia cells. (**** = *p* < 0.0001).

Kaplan-Meier curves were generated for survival studies and analyzed by logrank tests. Single-factor analyses of abnormal BCP cell counts were performed using student’s *t*-tests, while statistics for comparisons of means were performed using one- or two-way ANOVAs as indicated. Post-hoc testing for multiple comparisons was performed as indicated. See Supplemental Material for details of statistical analysis of data presented in Fig. 1B. Statistical analyses were performed using Prism 5 for Mac OS X (GraphPad, San Diego, CA). Where indicated, *p < 0.05, **p < 0.01, ***p < 0.001, ****p < 0.0001.

## Results

### Leukemia in Eμ-Ret mice emerges from an early-occurring abnormal BCP cell population

As described previously, Eμ-Ret mice express an oncogenic *Ret* fusion gene under the control of the IgH enhancer [26, 27], which generates an abnormal fetal BCP population with leukemia-initiating activity (hereafter called preleukemic) [28]. Consistent with an in utero initiation event, an expanded Hardy Fraction C pro-B cell population [36], defined by a characteristic B220^int^/CD43^int^/BP-1^hi^ phenotype, is detectable in blood, spleen, and bone marrow (BM) of young Eμ-Ret mice (Fig. 1A, Supplemental Fig. 1). No similar population is detectable in transgene-negative littermates. The size of the preleukemia population varies substantially between young mice (Fig. 1B) and does not exhibit consistent expansion over the first months of life (Fig. 1C); significantly elevated numbers of the abnormal BCP cells in peripheral blood correspond with onset of overt leukemia. Combined survival studies involving 182 mice housed in SPF-free facilities in either Vancouver or Philadelphia for the entirety of their lives demonstrated a broad age-of-onset range (median leukemia-free survival, 155 days), with near 100% penetrance after one year on the BALB/c background (Fig. 1D). Six deaths unrelated to leukemia progression occurred in the study. Presentation of leukemia is associated with splenomegaly and lymphadenopathy (Fig. 1E and Supplementary Fig. 2), resulting from a massive infiltration of blasts with the characteristic immunophenotype (Fig. 1F, G).

To confirm that the early-occurring abnormal BCP cell population was sufficient to generate leukemia, we purified these cells from spleens of 2-week-old Eμ-Ret mice and transplanted 1–3 × 10^5^ cells into non-preconditioned BALB/c or NSG mice. Recipient mice were then monitored for disease onset. NSG recipients developed overt leukemia after a variable latency (60–150 days, Fig. 1H, left panel), which is similar to the range of latency observed in Eμ-Ret mice. In contrast, no leukemia arose in BALB/c mice engrafted with preleukemic BCP cells. However, BCP blasts isolated from moribund NSG recipients of preleukemia quickly progressed to full blown disease in secondary BALB/c recipients (Fig. 1H, right panel), consistent with the rapid outgrowth observed in BALB/c mice engrafted with primary leukemia from Eμ-Ret mice [37, 38]. This result confirms that the early-arising preleukemic BCP population is sufficient for leukemia generation and indicates that the engraftment and/or progression of preleukemia may be influenced by the immune status of the recipient mice.

### Eμ-Ret leukemia is characterized by non-random chromosomal gains

**Fig. 2:**
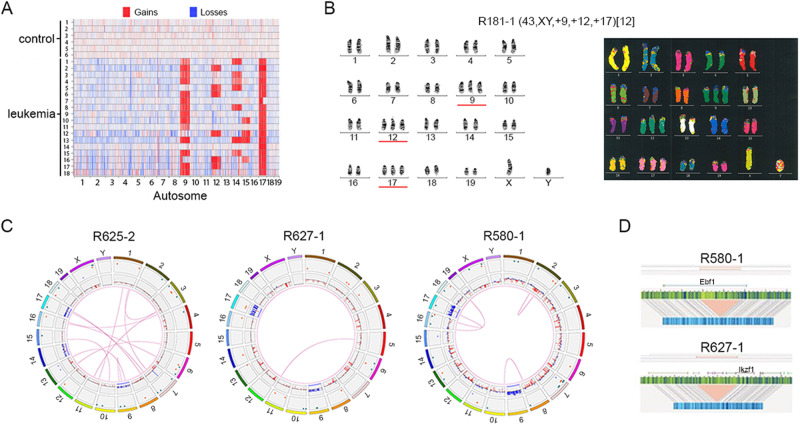
Eμ-Ret leukemia displays non-random chromosome gains. **A** CMA analysis reveals a non-random pattern of chromosome gains in Eμ-Ret leukemia, involving combinations of chromosomes, 9, 12, 14, 15, and 17. **B** Representative G-banding (from 8 total, left panel) and M-FISH (from 4 total, right panel) karyotypes for primary Eμ-Ret leukemia sample (R181-1) showing trisomy of chr 9, 12, and 17. No large SVs are detectable. **C** Circos plots for 3 primary Eμ-Ret leukemia samples reveal the presence of sample-specific chromosome gains, deletions, insertions, duplications and translocations, most occurring at a sub-clonal level. **D** Genome mapping of monoallelic deletions involving *Izkf1* and *Ebf1* in independent samples, both at a VAF of 0.46.

Chromosome microarray analysis (CMA) of spleen samples from 18 moribund Eμ-Ret mice revealed that each leukemia carried additional chromosomes, with total content ranging from 41 to 44 (median, 43) (Fig. 2A). Chromosomal gains were non-random, with additional copies of chromosome (chr) 9, 12, 14, 15, and 17 detected in multiple samples; chr 17 in all samples, chr 9 in 15, chr 14 in 7, chr 12 in 6, and chr 15 in 4. No whole-chromosome losses were detected in any leukemia sample. G-Banding analyses of two additional Eμ-Ret leukemia samples revealed that the additional chromosomes generated trisomies of chr 9, 12, and 17 (Fig. 2B, Supplementary Fig. 3, and Table 1). M-FISH analysis validated the G-Banded karyotype and confirmed the absence of large inter-chromosomal rearrangements in sample R181-1 (Fig. 2B, right panel).

In addition to whole chromosome gains, many deletions and duplications were detected by CMA. To investigate structural variants (SV) at higher resolution, we evaluated three additional Eμ-Ret leukemia samples by optical genome mapping (OGM). After subtraction of variants detected in transgene-negative controls, OGM confirmed hyperdiploidy involving combinations chromosomes 9, 14, and 17 in each sample (Fig. 2C), with sample R627-1 containing two additional copies of chr 17. In addition to chromosomal gains, other SVs were detected in each sample by OGM, but these were sample-specific and generally present at sub-clonal variant allele frequency (VAF; range 0.04 – 0.54), indicating the presence of multiple subclones. Notably, deletions involving two key B lineage transcription factors were detected in 2 of the 3 samples at VAF of ~0.5; a deletion of the 3′-half of *Ebf1* (sample R580-1) and a deletion involving the 5′-end of *Ikzf1* (sample R627-1) (Fig. 2D). Such monoallelic deletions are consistent with secondary genomic alterations detected in human HD-ALL [5]. Confirmation that the deletion in *Ebf1* led to reduced expression in sample R580-1 was obtained by qPCR (Supplementary Fig. 4). In the case of *Ikzf1*, reduced expression was detected in all three leukemia samples, compared to BCP cells from transgene-negative BALB/c mice. This result was, therefore, unable to validate the *Ikzf1* focal deletion, but implicated reduced *Ikzf1* expression in Eμ-Ret leukemogenesis.

Mutations in HD-ALL are generally low in number and sub-clonal, and none has been found to be common to all patients. To identify whether the Eμ-Ret leukemias also lack shared driver mutations, we performed whole-exome sequencing (WES) on preleukemic BCP (*n* = 5) and overt leukemic (*n* = 5) samples (Supplementary Table 1). Within this small sample cohort, Eμ-Ret HD-ALL was found to harbor similarly low numbers of SNV. Although some variants were recurring, no mutation was found in all samples and none involved genes commonly linked to human B-ALL.

### Hyperdiploidy is an early event in Eμ-Ret leukemogenesis

**Fig. 3:**
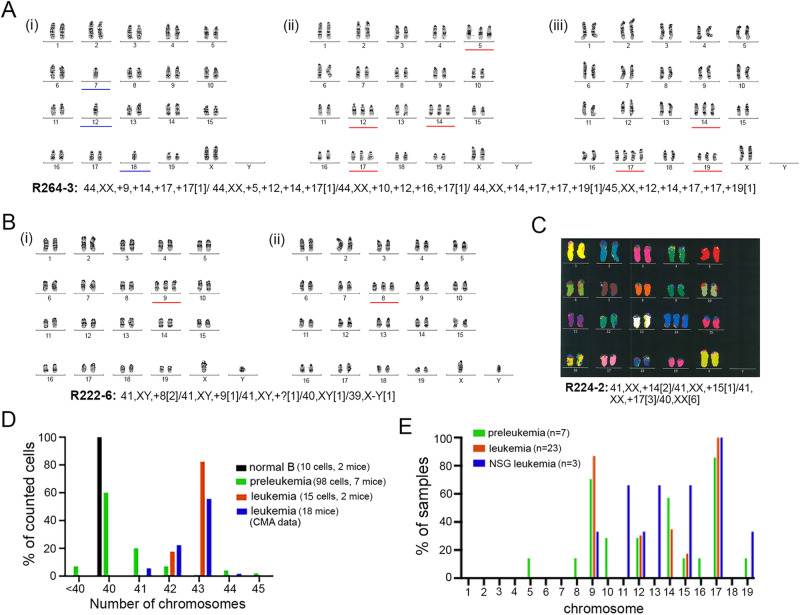
Aneuploidy is an early event in Eμ-Ret B-ALL progression. Representative G-banded karyotypes within the splenic preleukemic cell population of healthy Eμ-Ret mice, R222-6 (**A**) and R264-3 (**B**) showing chromosomal heterogeneity within the preleukemic cell population. Full reported karyotype is shown for each sample. **C** An M-FISH karyotype (from 5) for preleukemic sample (R224-2) revealing the presence of trisomy 14 and lack of large SVs. Full karyotype is provided below the image. **D** Range of chromosome numbers in normal splenocytes (10 cells, 2 mice), purified preleukemic cells (98 cells, 7 mice), and leukemic cells (17 cells, 2 mice) detected by G-banded karyotype analysis. Numbers detected in leukemia samples by CMA (18 mice) is shown in blue for comparison. **E** Heterogeneity of chromosomal involvement in preleukemic (*n* = 7), leukemic (*n* = 23, including both G-banded and CMA samples), and leukemia arising in NSG recipients (*n* = 3).

The generation of aneuploidy is the earliest detectable abnormality in human HD-ALL [4, 16]. To determine if the acquisition of additional chromosomes was a similarly early occurrence in Eμ-Ret leukemogenesis, we performed G-banded karyotyping on 7 preleukemic cells samples purified from spleens of 2-week old Eμ-Ret mice (Table 1). In addition to combinations of trisomy 9, 14, and 17, extra copies of chromosomes not observed in leukemia samples (i.e. chr 5, 8, 10, 16, and 19) were also detected (Fig. 3A, B and Table 1). Although uncommon, loss of chromosomes was also observed in preleukemic samples (Fig. 3A). As a result, most preleukemia karyotypes were more complex than those of leukemia. Consistent with findings from leukemia cells, large inter-chromosomal translocations were not apparent in the one preleukemic sample (R224-2) analyzed by M-FISH (Fig. 3C).

**Table 1:** Karyotypes of normal, leukemic and leukemia-initiating Eμ-Ret-derived B cells.

Sample type	ID	# analyzed	# normal	# HyperD	# HypoD	Karyotype
Control B cells	224-2	5	5			40,XX[5]
Control B cells	224-6	5	5			40,XX[5]
Eμ-Ret Leukemic	181-1	14	0	14	0	43,XY, +9, +12, +17[12]
Eμ-Ret Leukemic	186-6	3	0	3		42,XY, +9, +17[3]
Eμ-Ret preleukemia	264-2	30	29		1	40,XY[30]
Eμ-Ret preleukemia	264-3	30	18	7	5	44~45,XX, +5, +9, +10, +12, +14, +16, +17, +17, +19[cp5]
Eμ-Ret preleukemia	214-16	5	1	4	0	41~43,XX, +9, −10, +14, +17,+mar[cp4]
Eμ-Ret preleukemia	216-14	4	0	4	0	42,XY, +9, +14, +17[cp3]
Eμ-Ret preleukemia	222-6	6	1	4	1	41,XY, +8, +9,+mar[cp4]/40,XY[2]
Eμ-Ret preleukemia	224-2	13	7	6	0	41,XX, +14, +15,17[cp6]/40,XX[6]
Eμ-Ret preleukemia	224-6	13	3	10	0	41~42,XX, +9, +12, +17[cp10]/ 40, XX[3]
NSG Leukemic	N-R6-4	10	0	10	0	41~43,XY, +9, −11, +13, +15, +17, +19[cp9]
NSG Leukemic	N-R6-1	10	1	9	0	42,X,-Y, +12, +15, +17[cp9/40,XY[1]
NSG Leukemic	N-R6-3	9	0	9	9	42~43,XY, −11, +13, +17, +17,+mar1, +mar2[cp9]

To confirm that aneuploidy was restricted to the abnormal BCP population in preleukemic mice, mature B cells and preleukemic BCP cells were purified from the same mouse spleen (R224-6) prior to analysis. In contrast to the consistently diploid mature B cells, the majority of preleukemia cells analysed were aneuploid (Table 1 and Supplementary Fig. 5). Overall, preleukemia samples were found to harbor a broader range of chromosome number (Fig. 3D) and more diversity of chromosomal involvement (Fig. 3E) than leukemia samples. Preleukemia samples harbored the occasional hypodiploid cells and many euploid cells, while leukemic aneuploidy clustered around 2–3 additional chromosomes in both manual karyotyping and CMA analyses. The finding that preleukemia is associated with more chromosomal diversity than leukemia suggests disease evolution from a pool of heterogeneous preleukemic cells.

As preleukemic cells do not progress to leukemia in BALB/c (Fig. 1H) and immune surveillance has been reported to exert selective pressure on aneuploid cells [39], we evaluated the chromosomal heterogeneity in the leukemias arising after transfer of preleukemic BCP populations into immune-deficient NSG mice. While extra copies of chr 9 (1 of 3 samples), chr 12 (1 of 3), chr 15 (2 of 3), and chr 17 (3 of 3) were again present, trisomies of chr 13 (2 of 3 samples) and chr 19 (1 of 3 samples) were also detected (Table 1). In addition, loss of one copy of chr 11 was observed in 2 of the 3 samples. Overall, NSG-derived leukemias exhibited chromosomal heterogeneity more similar to preleukemia than to primary leukemia samples from Eμ-Ret mice (Fig. 3E and Table 1). This result suggests that reduced immune-mediated selective pressure may allow the progression of less “fit” aneuploid subpopulations.

### RNA-seq reveals shifts in pathway utilization after preleukemia to leukemia transition

**Fig. 4:**
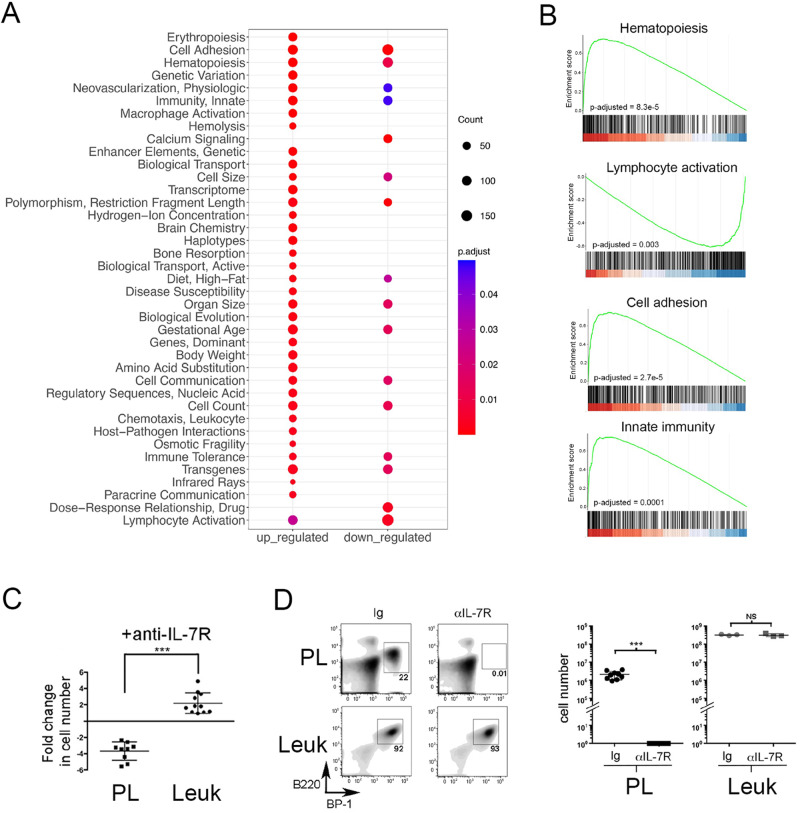
Cell pathway changes associated with transition to overt leukemia. **A** The most significantly enriched pathways (MeSH terms), either up-regulated or downregulated in leukemia cells compared to preleukemia, identified by GSEA (FDR < 0.05). **B** GSEA plots depicting gene enrichment in selected pathways. Adjusted *p* values for multiple testing are shown. **C** Preleukemic cells isolated from healthy 4-week-old Eμ-Ret mouse spleens (PL) or splenic leukemia cells (Leuk) from moribund Eμ-Ret mice were cultured in the presence or absence anti-IL-7R antibody and their fold expansion/depletion calculated as the number of viable cells at 72 h divided by the number seeded into culture (fold change of preleukemia (*n* = 9) vs. leukemic cells (*n* = 12), *p* = 0.012). **D** Splenic BCP cell burden in NSG recipients of preleukemic or leukemic cells, 14 days after treatment with anti-IL7Rα or control antibody. Left panel shows density plot for one representative NSG mouse from each group; right panel shows cell numbers recovered from each recipient NSG mouse from 4 independent preleukemia population samples and one representative leukemic cell sample. For Ig vs. αIL-7R, *p* < 0.0001 for preleukemia and *p* = 0.7 for leukemic cells (*p* values were calculated using student’s *t*-test; NS not significant).

To identify cellular pathways involved in the transition from preleukemia to leukemia, we compared gene expression profiles between preleukemic BCP cells isolated from young, healthy Eμ-Ret mice and leukemia cells from moribund older mice. PCA analysis of RNA-seq data revealed that preleukemia and leukemia samples were readily clustered, with preleukemia samples bearing greater similarity than leukemia samples (Supplementary Fig. 6). This finding suggests that transformation may result from dysregulation of various pathways. Expression of many genes associated with human B-ALL was significantly altered between preleukemic and leukemic BCP cells (Supplementary Table 2A, B), including down-regulation of *Pax5*, *Blk*, *Cdkn2a*, *Igll1*, *Tcf3*, and *Vpreb1*. The reduced expression of Pax5 was consistent with the observed down-regulation of several Pax5 target genes in leukemic cells, including *Ebf1*, *Bst1*, *Cd19*, *Cd79a*, *Ccnd3*, *Vpreb3*, and *Nedd9* [40]. GSEA implicated the dysregulation of multiple pathways in the transformation to leukemia (Fig. 4A), including those associated with hematopoiesis and lymphocyte activation, and pathways previously reported to be affected in HD-ALL (Fig. 4B) [25].

To identify the functional impact of gene expression changes, we compared the growth factor dependency of preleukemia and leukemia cells. In vitro, preleukemia cells from healthy Eμ-Ret mice declined in number when cultured in the presence of IL-7 receptor-blocking antibody, consistent with the requisite role of IL-7 for mouse BCP cell survival (Fig. 4C) [41]. In contrast, leukemic blasts obtained from sick mice expanded in number under the same culture conditions. This switch in growth factor dependency was confirmed in vivo, where the acquired IL-7-independence of leukemia cells was sufficient to sustain the expansion of leukemic blasts in NSG recipients treated with IL-7R-blocking antibody (Fig. 4D). The same blocking-antibody treatment eliminated preleukemic cells transplanted into NSG mice. These results confirm and extend on previous reports that changes in cytokine responsiveness are associated with leukemia progression in Eμ-Ret mice [27].

### Hyperdiploidy induces centrosome amplification in Eμ-Ret leukemia cells

**Fig. 5:**
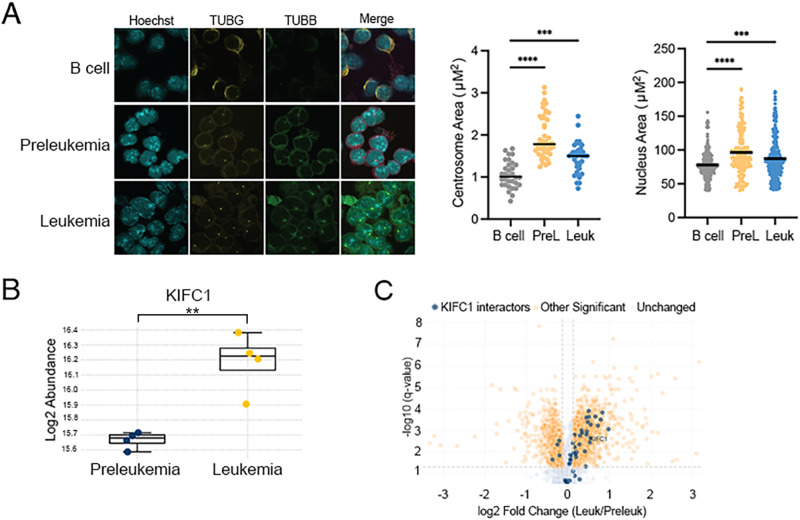
Centrosome phenotype in mouse splenocytes and aneuploid B-ALL cells. **A** Immunofluorescence analysis of primary B cell, preleukemia, and leukemia samples grown in ex vivo co-culture with stromal cells. Cells were stained with antibodies recognizing gamma-tubulin (TUBG) and beta-tubulin (TUBB) and counterstained with the nuclear dye DAPI. Quantification of centrosome area (*n* ≥ 30 cells) and nucleus area (*n* ≥ 200) was performed for each sample. Mass spectrometry analysis indicates differential protein abundance of **B** KIFC1 and **C** proteins known to interact with KIFC1 (highlighted with blue on Volcano plot) between preleukemia and leukemia samples sorted from Eμ-Ret mice (*n* = 4 for each). (** = *p* < 0.01, *** = *p* < 0.001; **** = *p* < 0.0001).

As the generation of aneuploidy is strongly correlated with the presence of centrosome amplification (CA), we quantified centrosome area in normal B cells, preleukemia cells, and leukemia cells using TUBG staining. As we noted a very small phospho-histone H3-positive mitotic population in both preleukemia and B cell samples, we examined centrosome areas in non-mitotic cell populations, using a protocol modified from our previously published work [42]. We found significant alterations in centrosome area and nucleus area in preleukemia and leukemia samples (Fig. 5A). In addition, we measured centrosome amplification in mitotic and interphase BALB/c splenocytes and Eμ-Ret leukemia cells, using co-staining of pericentriolar material (pericentrin), internal centriole (centrobin) and microtubules (tubulin), to define centrosomes. All splenocytes contained either one or two centrosomes with normal size (Supplementary Fig. 7A). In contrast, centrosomes in Eμ-Ret leukemia cells exhibited either size (larger than 4 μm^2^) or numerical (>2 centrosomes per cell) abnormalities. Moreover, fragmentation of pericentriolar material, which is commonly seen in cancer cells [43], was also observed in mouse leukemic cells (Supplementary Fig. 7A). While the levels of abnormal centrosomes (size and number) detected in either interphase and mitotic splenocytes were low, close to half of the mitotic Eμ-Ret leukemia cells contained abnormal centrosome phenotypes (Supplementary Fig. 7B).

To maintain viability during mitosis, aneuploid cells adapt by clustering their extra centrosomes, a process that requires the kinesin-14 family protein KIFC1 [44–46]. As Eμ-Ret leukemia represents the emergence of a viable, consistently hyperdiploid cell population from a more numerically diverse aneuploid preleukemic cell pool, we hypothesized that KIFC1 would be enriched in leukemic samples. To investigate this, we performed proteomic analysis of sorted preleukemia and leukemia samples (*n* = 4 for each). KIFC1 was detected at significantly higher abundance in leukemic samples (Fig. 5B). Further supporting a role for this pathway in Eμ-Ret leukemia progression, several proteins known to interact with KIFC1 were similarly upregulated in leukemia samples (Fig. 5C).

### Targeting abnormal centrosomes selectively depletes the abnormal Eμ-Ret BCP cells

**Fig. 6:**
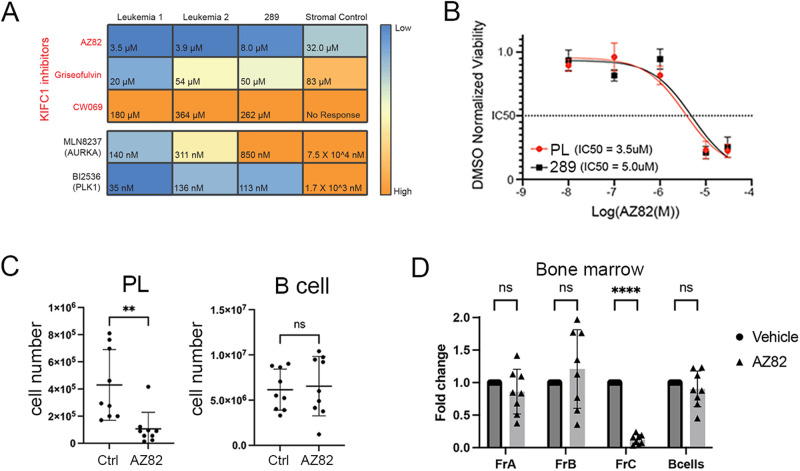
Depletion of abnormal Eμ-Ret BCP by targeting centrosome amplification. **A** Heatmap depicting the IC50 concentrations for KIFC1 inhibitors (AZ82, Griseofulvin, and CW069), AURKA inhibitor (MLN8237), and PLK1 inhibitor (BI2536) against two primary Eμ-Ret leukemia samples, the Eμ-Ret-derived 289 cell line, and stromal cells (control). IC50 values represent the mean of 3 replicate experiments. **B** Cell viability normalized to DMSO control in primary preleukemia (PL) and 289 cell line cell cultures treated for 72 h with serial dilutions of AZ82. IC50 value was determined with a linear regression curve, and is indicated by the dashed line (mean ± SEM, *n* = 6 replicates). **C** Preleukemia and normal B cell numbers in spleens from healthy adult Eμ-Ret mice treated with AZ82 or control. **D** BCP cell (Hardy fractions A, B and C), and B cell subset numbers in bone marrow from adult Eμ-Ret mice treated with AZ82 or control. (For **C**, **D**, results shown a mean ± SD, *n* = 9 for both). **p < 0.01, ****p < 0.0001.

Reliance on centrosome clustering for viable cell division potentially renders aneuploid cells sensitive to inhibitors of proteins required for handling CA, including KIFC1 [47–51]. Therefore, we assessed the impact of KIFC1 inhibitors [52], as well as inhibitors of the mitotic kinases, aurora kinase A (AURKA) and polo-like kinase 1 (PLK1), on 2 primary Eμ-Ret leukemia samples and the 289 cell line. AURKA and PLK1 both have essential roles in mitotic spindle assembly and integrity and have been experimentally associated with a centrosome amplification gene signature (Supplementary Fig. 8) [53]. For KIFC1 inhibitors, we found that both primary leukemia samples showed a higher sensitivity to AZ82 (3.5 μM, 3.9 μM) in comparison to Griseofulvin (20.2 μM, 54.4 μM) and CW069 (363.7 μM, 261.5 μM) (Fig. 6A). Stromal cells were relatively insensitive to these agents. Eμ-Ret-derived cells were also highly sensitive to the PLK1 inhibitor (BI2536) and AURKA inhibitor (MLN8237) (Fig. 6A).

As centrosome abnormalities are present in Eμ-Ret preleukemia cells, we hypothesized that the preleukemic population would be similarly sensitive to the inhibitor drugs. In AZ82 titration assays, the IC50 for preleukemic cells was comparable to that of 289 cells (Fig. 6B). Consistent with this in vitro sensitivity, i.p. administration of three doses of AZ82 or vehicle to 9 healthy, 5–7-week-old Eμ-Ret mice led to a significant depletion of preleukemic BCP cells, but not normal B cells, in the spleens of treated mice (Fig. 6C). Furthermore, analysis of BCP subpopulations in the bone marrow of these mice demonstrated that AZ82 achieved selective depletion of Fraction C, the late pro-B cell compartment dominated by preleukemic BCP cells in Eμ-Ret mice (Fig. 6D).

## Discussion

The Eμ-Ret mouse was one of the first transgene-driven models of BCP leukemia/lymphoma, providing early support for a two-hit pathway of B-ALL progression and modeling its frequent origin during fetal development [26–28]. In this study, we extend the description of Eμ-Ret leukemogenesis by demonstrating the emergence of leukemia bearing non-random chromosomal gains from a heterogenous pool of aneuploid preleukemic BCP cells. All trisomies in leukemia cells involved chromosomes 9, 12, 14, 15, or 17. While a single chromosome gain was frequently observed in preleukemic cells, the majority of leukemia samples possessed 2–4 trisomies. As with pediatric HD-ALL, chromosome losses occur less frequently, but were observed in karyotypic analyses. Hyperdiploidy was accompanied by centrosome amplification, which rendered both preleukemia and leukemia cells sensitive to inhibitors of centrosome clustering and multipolar spindle formation. Overall, these results establish the Eμ-Ret mouse as a novel model for investigating the genesis, selection, and outgrowth of this poorly understood pediatric B-ALL subtype.

A change in chromosome number is the only abnormality common to all HD-ALL patients and is often the only recognized leukemia-promoting aberration shared between diagnostic and relapse clones in individual patients [3, 19]. For these reasons, targeting the molecular adaptations that sustain hyperdiploid cell viability represents a rational strategy to improve outcomes for the HD-ALL patient population by further depleting the residual, often ancestral, clones that drive relapse. Centrosome amplification has long been regarded as a promising therapeutic target for aneuploid cancer cells [51]. We, and others, have shown that ALL cells show a high prevalence of centrosome amplification and are sensitive to agents that interfere with centrosome clustering [30, 54]. Here, our results significantly expand on those findings by demonstrating that a hyperdiploid preleukemic cell population is sensitive to inhibitors of centrosome clustering in vivo. This finding provides strong evidence that drugs targeting centrosome amplification could reduce the incidence of relapse by depleting the ancestral clones that drive disease recurrence in the majority of patients.

The majority of hyperdiploid karyotypes in HD-ALL are thought to result from a single aberrant mitotic event, which results in a clone that remains relatively stable through disease progression. However, deviations from the modal chromosome number have been reported in diagnostic HD-ALL samples, suggesting a possible role for chromosomal instability in disease development [7, 8, 11, 19]. The observation that Eμ-Ret preleukemia comprises a range of aneuploidy suggests that the heterogeneity of HD-ALL may reflect the differential progression rates of independently generated, long-lived preleukemic clones. Our data are consistent with an initiating abnormal mitotic event producing preleukemic populations with randomly generated whole-chromosome gain(s) or loss(es). Individually, these aneuploid genotypes induce selective pressures, such as an intrinsic negative selection due to gene dosage effects [55] or extrinsic selection imposed by immune surveillance. Our findings support that certain chromosome configurations (e.g. +17, +9 in Eμ-Ret mice) are more advantageous for leukemogenesis, while most aneuploid combinations detected within preleukemic BCP cells are likely detrimental and lead to a failure to overcome the intrinsic and extrinsic pressures. Changes in the strength or nature of the selective pressures, such as the immune deficiency of NSG mice, may enable the progression of clones that would be otherwise unlikely to survive.

The cellular mechanism that generates the initial aneuploidy in human B-ALL remains unknown. In the case of Eμ-Ret mice, the *Ret* fusion gene is the driver of HD-ALL. Although sub-clonal mutations in *Ret* have been reported for the iAMP21 B-ALL sub-type [16], it has not been implicated in HD-ALL and mechanisms through which it could enable aneuploidy remain unknown. While aneuploidy is not a hallmark of Ret-driven malignancies, the RET/PTC fusion protein has been shown to generate nuclear envelope irregularity [56], which has been linked to genome instability [57, 58]. Intriguingly, the single reported B-ALL patient with a *RET* gene fusion presented with a near tetraploid karyotype [59]. It will be of interest to determine if Eμ-Ret transgene expression generates nuclear envelope changes or other abnormalities detected in human HD-ALL blasts [9, 10], in BCP cells. A perhaps more likely mechanism by which the *Ret* transgene could enable HD-ALL leukemogenesis is by establishing tolerance to aneuploidy in BCP cells, leading to the formation of a pool of preleukemic cells that would otherwise have been eliminated [60]. Interestingly, Stat3 activation, which is induced by Ret signaling [61], has been reported to regulate centrosome clustering [62]. Other downstream effects of oncogenic Ret signaling could also contribute to the generation of a tolerant phenotype [63]. Understanding the precise nature of the transgene-mediated pro-leukemic activities will be essential to determine which aspects of human HD-ALL leukemogenesis are most accurately modelled in Eμ-Ret mice.

Second hit mutations identified in HD-ALL are generally sub-clonal and heterogenous, with even those affecting the Ras pathway occurring in only ~30% of HD-ALL patients [22, 24]. Eμ-Ret leukemia similarly lacks a characteristic mutation signature. Structural deletions affecting *Ebf1* and *Ikzf1*, two lymphoid transcription factors identified as frequent targets of second hit mutations in pediatric B-ALL [2, 5], suggest shared leukemogenic processes between human and murine HD-ALL. Consistent with this hypothesis, transition from preleukemia to leukemia in Eμ-Ret mice was marked by changes in expression of many genes implicated in human B-ALL, resulting in alteration of an similar array of cellular pathways [9, 10]. The notable absence of Ras pathway mutations in Eμ-Ret leukemia may reflect the oncogenic activity of the *Ret* transgene. Ret fusion proteins have been shown to activate several downstream signaling pathways, including Ras, in solid tumours and leukemia [61, 64]. In medullary thyroid cancer, *RET* and *RAS* driver mutations are mutually exclusive [65], and a similar relationship may exist in Eμ-Ret B-ALL.

The presence of additional chromosome 17 copies in all Eμ-Ret leukemia samples echoes the usual occurrence of multiple copies of chromosome 21 in pediatric HD-ALL and may reflect an essential gene dosage effect. While mouse chr 16 is generally regarded as the homolog for human chr 21 based on syntenic regions, trisomy 16 is associated with reduced competitive fitness of HSCs from mouse fetal liver [60]. Human chromosome 21 also shares an orthologous region with mouse chromosome 17, which has been identified as a major determinant of Down syndrome-related developmental cognitive deficits [66]. A role for this region in leukemogenesis has yet to be reported, but the restricted and reproducible pattern of numerical changes observed in Eμ-Ret leukemia may provide the means for identifying the critical interactions that generate, select, and sustain aneuploid B cell precursors.

Overall, these results establish Eμ-Ret mice as the first experimental model of HD-ALL, the most common pediatric malignancy. Further, the study demonstrates the utility Eμ-Ret mice for investigating novel strategies to deplete leukemia-initiating and -propagating cells in a consistent model of B-ALL bearing non-random chromosomal gains. Our findings suggest considerable shared biology that could enable a detailed unveiling of the HD-ALL leukemogenesis and clonal evolution, including the role of immune surveillance mechanisms. Such investigations could reveal novel vulnerabilities associated with hyperdiploidy and supernumerary chromosomes that could be targeted for treatment of the significant number of HD-ALL patients who suffer a relapse despite the generally good prognosis associated with this leukemia.

### Supplementary information

Supplemental Material

## Data Availability

RNA-seq data from this study has been deposited in the NCBI GEO repository (accession number GSE257537). Other datasets generated and/or analysed during the current study are available from the corresponding author on reasonable request.
