# Supplementary material for: The Eμ-Ret mouse is a novel model of hyperdiploid B-cell acute lymphoblastic leukemia
Source: Leukemia. 2024 Mar 22;38(5):969–80. doi: 10.1038/s41375-024-02221-x (PMC11073968; doi:10.1038/s41375-024-02221-x)
Supplement: Supplementary file 1 — Supplemental Material [file 41375_2024_2221_MOESM1_ESM.docx]

**Supplemental Material**

**Additional Methods**

**Real-Time Reverse Transcriptase-Polymerase Chain Reaction**

Quantitative reverse transcriptase-polymerase chain reaction (qRT-PCR) was used to measure *Ikzf1* and *Ebf1* gene expression in the leukemic samples subject to OGM analysis. Leukemia sample R625-2 was used as the baseline to detect for changes in gene expression resulting from genomic deletions. RNA extraction was performed using RNeasy plus mini kit (Qiagen), according to the manufacturer’s protocol. For cDNA synthesis 200 ng of total RNA and SuperScript IV VILO cDNA synthesis master mix kit with ezDNase (Thermo Fisher Scientific™) was used. Real-Time PCR was performed in duplicate using the QuantStudio™ 5 Instrument (Thermo Fisher Scientific™) and SYBR Green PCR master mix for detection. Primer sequences are as following: *Ikzf1*-F-5’-AAGAGCGATGCCACAACTAC-3’, *Ikzf1*-R-5’-GTCTTCTGCCATCTCGTTGT-3’, *Ebf1*-F-5’-TCGTACAAGTCCAAGCAGTTC-3’, *Ebf1*-R-5’-GGAATGACCTTCTGTAACCTCTG-3. Relative expression of the target genes was calculated by 2 ^–∆∆Ct^method and *Gapdh* was use as a reference gene.

**Whole Exome Sequencing**

Exome library preparation was performed using the Agilent SureSelectXT Mouse All Exon Kit. Purification of the captured library were achieved by binding to MyOne Streptavidin T1 Dynabeads and enriched via off-bead PCR amplification in the linear range. Sequencing (2x100 bp) was performed with a 6-bp index read using the TruSeq SBS Kit v3 on an Illumina HiSeq 2500. Fastq files were generated through BcltoFastq 1.8.4. BWA version 0.7.4. was utilized to align the sequence data to the mouse reference genome (GRCm38.71). Conversion steps were carried out using SAMtools followed by the removal of duplicate reads. Local realignment around indels, SNP-calling, annotation, and recalibration was facilitated by GATK 2.4.9. Moreover, mouse dbSNP138 and dbSNP were used as training datasets for recalibration. The final variation calls were annotated by Variant Effect Predictor (VEP) using the Ensembl database (v70) and imported to a MySQL database for further analysis. Somatic calls were produced using MuTect (Cibulskis) and VarScan (Koboldt). VarScan2 filters were applied as suggested. Only results with at least 9% difference in allele frequency between sample and control were kept for further analysis.

**Karyotyping**

Cells were transferred to a THC (Trypsin-EDTA/Hypotonic/Colcemid) solution at a concentration of approximately 1million cells per mL. THC tubes were incubated at 37^o^C for 15 minutes and spun at 190 RCF for 10 minutes, followed by removal of the supernatant to a volume of 1 mL. Cells were re-suspended and a Carnoy’s fixative (3:1 methanol/acetic acid) was added drop-wise using a vortex, to avoid cell clumping. The process of centrifugation and fixation is repeated 2-3 times until a clean cell pellet is obtained. Metaphase chromosome preparations were then obtained by dropping fixed cell suspension on pre-cleaned microscope slides. Slides are manipulated to ensure optimal spreading of the chromosomes and then over-layered with fresh Carnoy’s fixative. Slides were aged from 1-3 days at room temperature prior to staining. Slides were stained using Trypsin-Giemsa Banding (1.5 mL Trypsin/ 50 mL EDTA Versene Buffer), and analyzed using Metasystems Ikaros software. Karyotypes were prepared for all analyzed cells.

**Proteomic analysis**

Prepared samples were processed for clean-up and trypsin digestion using SP3 through the addition of Sera-Mag Speed Beads. Briefly, the samples were mixed with SP3 beads mixture of hydrophilic and hydrophobic (1:1 ratio (v/v)) in 50mM HEPES, pH 8.0 and binding was initiated by the addition of nondenatured ethanol. After washing with 90% ethanol, proteins were digested with trypsin (1:50 ratio (v/v)) in 50mM HEPES, pH 8.5 at 37 °C overnight. The additional processing and cleanup of SP3-processed tryptic peptides was performed for subsequent MS analysis using SepPak C18 according to the manufacturer’s instructions.

Data was acquired by Data-independent acquisition (DIA) consisting of a MS1 scan from 300 to 1650 m/z (AGC target of 3e6 or 60 ms injection time), and resolution of 120,000 and a twenty-four-variable window format for MS2, (AGC target 3e6, resolution 30,000, auto for injection time), and stepped collision energy of 10% at 25%. Spectra were processed for identification, quantification and statistical analyses using Spectronaut with default settings. Two missed cleavages, carbamidomethyl cysteine was defined as fixed modification and methionine oxidation as well as N-terminal acetylation were defined as variable modifications. An FDR of less than 1% at peptide and protein level was enforced.

**Statistical Methods**

For Figure 1B, peripheral blood BCP cell burdens (expressed as number of BCP cells per ml of blood) were first log-transformed due to the skewed distribution. To evaluate the association between age (in days) and BCP cell counts, a piece-wise linear model was constructed with one slope before day 65 and another slope afterwards. Significant slopes would suggest BCP expansion is age-dependent. Peripheral blood percentages were also log-transformed and then analyzed using a linear mixed effects model was constructed to test the difference in the rates of change in peripheral blood percentages (log scale) over time between the two groups. The model included group, day, and group-by-day interaction as fixed effects, and included a random intercept and a random slope for each mouse. A significant group-by-day interaction would suggest that the outcome (log scale) changes at different rates for the two comparison groups.

Supplemental Table 1: SNV detected by WES

**Supplemental Table 1. SNVs detected in Eμ-Ret-derived abnormal BCP cells**

| **Gene** | **SNV** | **Leukemia**  **(out of 5)** | **Preleukemia**  **(out of 5)** | **Function** |
| --- | --- | --- | --- | --- |
| Cdh11 | Gly86Val  Val147Asp | 1  1 | 0 | Cell adhesion |
| Cps1 | Glu255Gly | 2 | 0 | Mitochondrial metabolism |
| Il1r1 | Ala71Val | 1 | 0 | Inflammation |
| Tnfsf14 | Gly64Arg | 1 | 0 | Immune response |
| Ifna14 | Ifna14 | 1 | 0 | Immune response |
| Prdx2 | Phe47Ile; Asp153His; Asp189Tyr | 1 | 0 | Antioxidant |
| Ifna6, | Thr145Lys  Val171Ile | 2  0 | 0  1 | Immune response |
| Ifna11 | Val171Ile | 2 | 1 | RNA splicing |
| Bud31 | Arg104Cys | 2 | 2 | RNA splicing |
| Mfsd5 | Ala346Val | 2 | 2 | Ion transport |
| Fus | Ser109Arg;Lys180Asn  Ser109Arg | 0 | 1  1 | Transcription |
| Rpsa | His131Arg  Ile159Met | 0 | 1  1 | Translation, adhesion |
| Rac1 | Ala144Val | 0 | 1 | GTPase |
| Fat4 | Ile3436Thr | 1 | 0 | Cell polarity |
| Tnfsf14 | Gly64Arg | 1 | 0 | Lymphoid activation |
| Lax1 | Pro67Ser | 1 | 0 | B cell activation |

**Supplemental Table 2A: Significantly down-regulated genes (FDR < 0.05, log2 fold change < 0)**

**
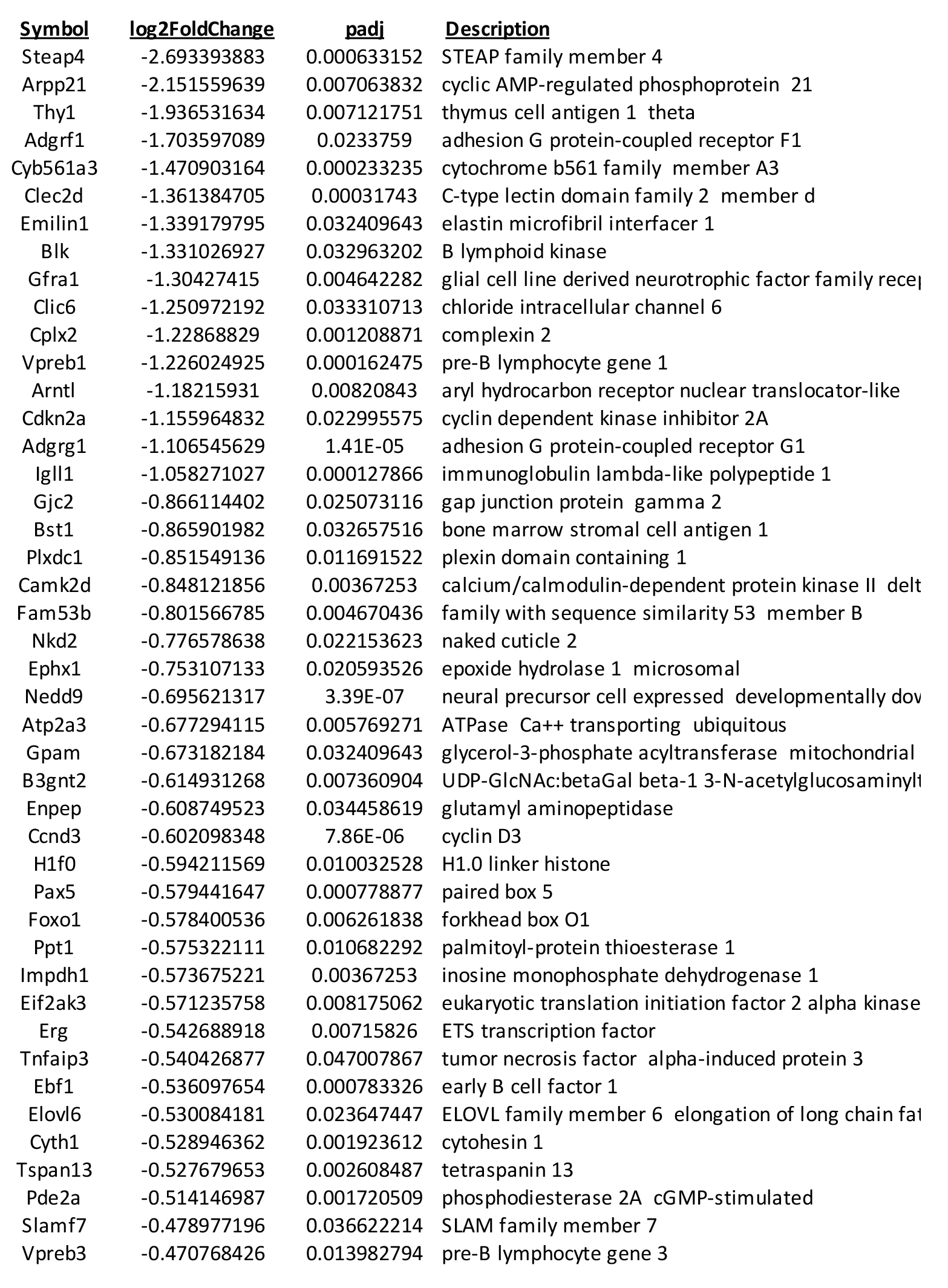
**

**Supplemental Table 2B: RNA-seq significantly up-regulated genes (FDR < 0.05, log2 fold change > 0)**


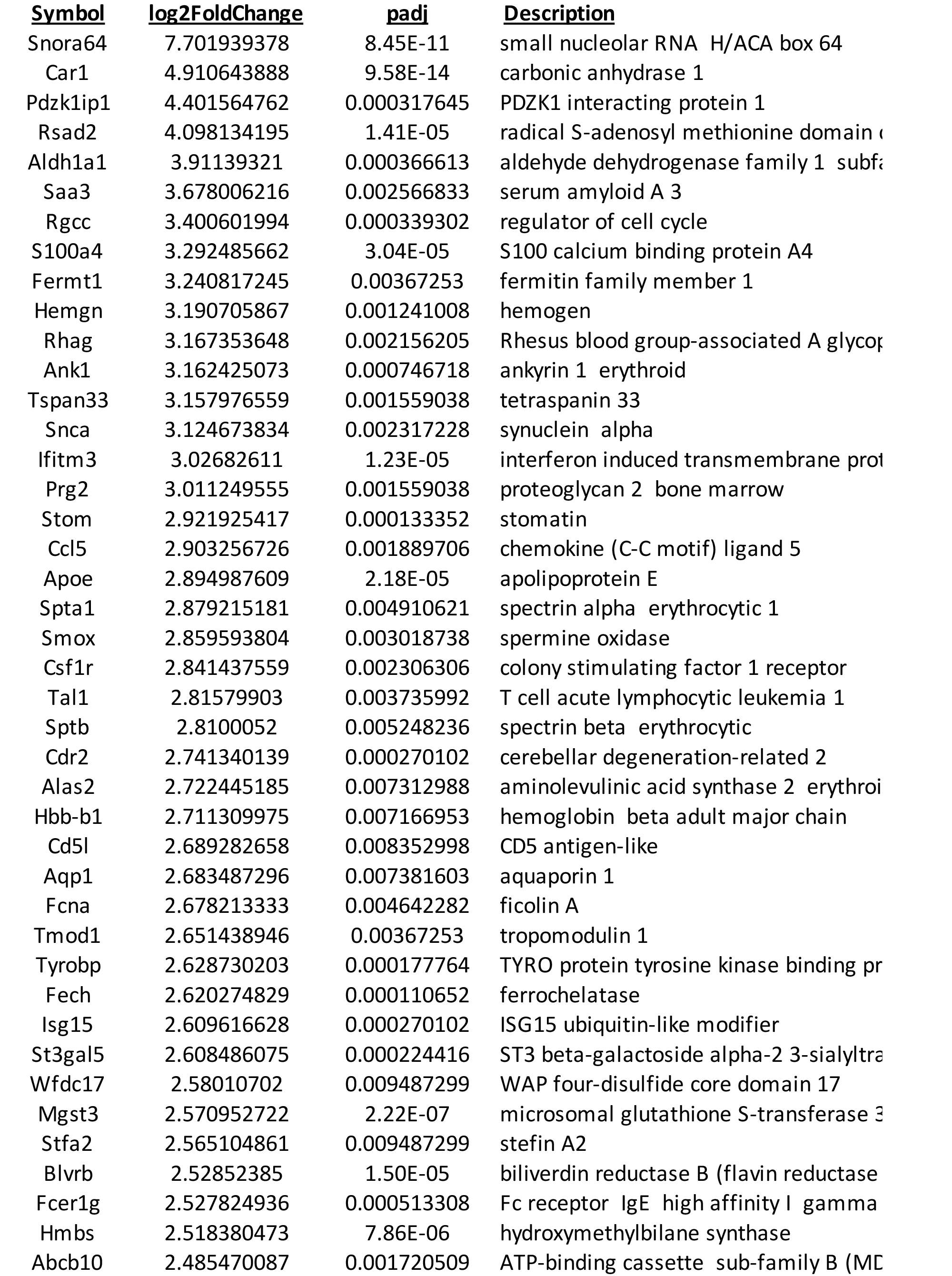


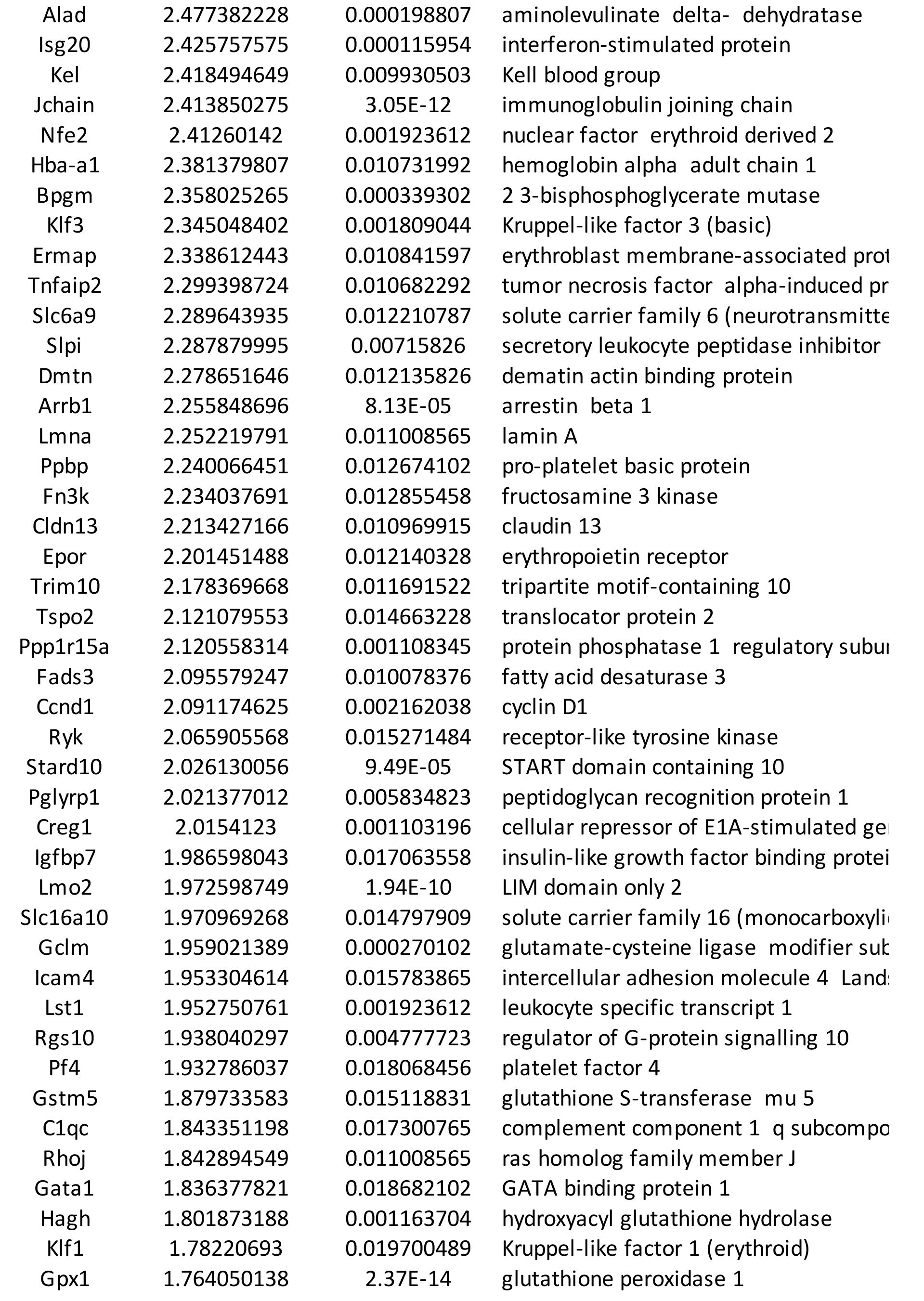


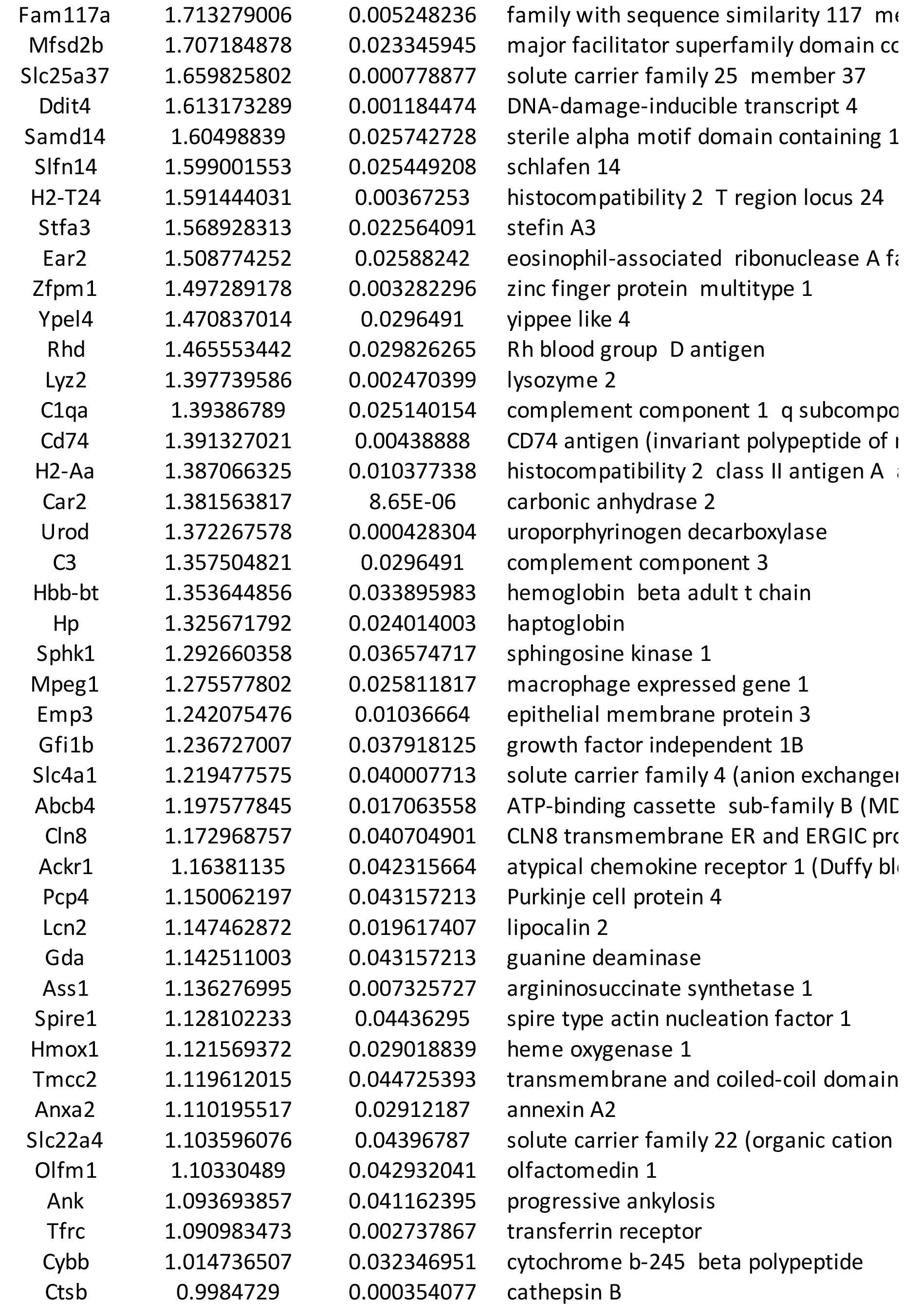


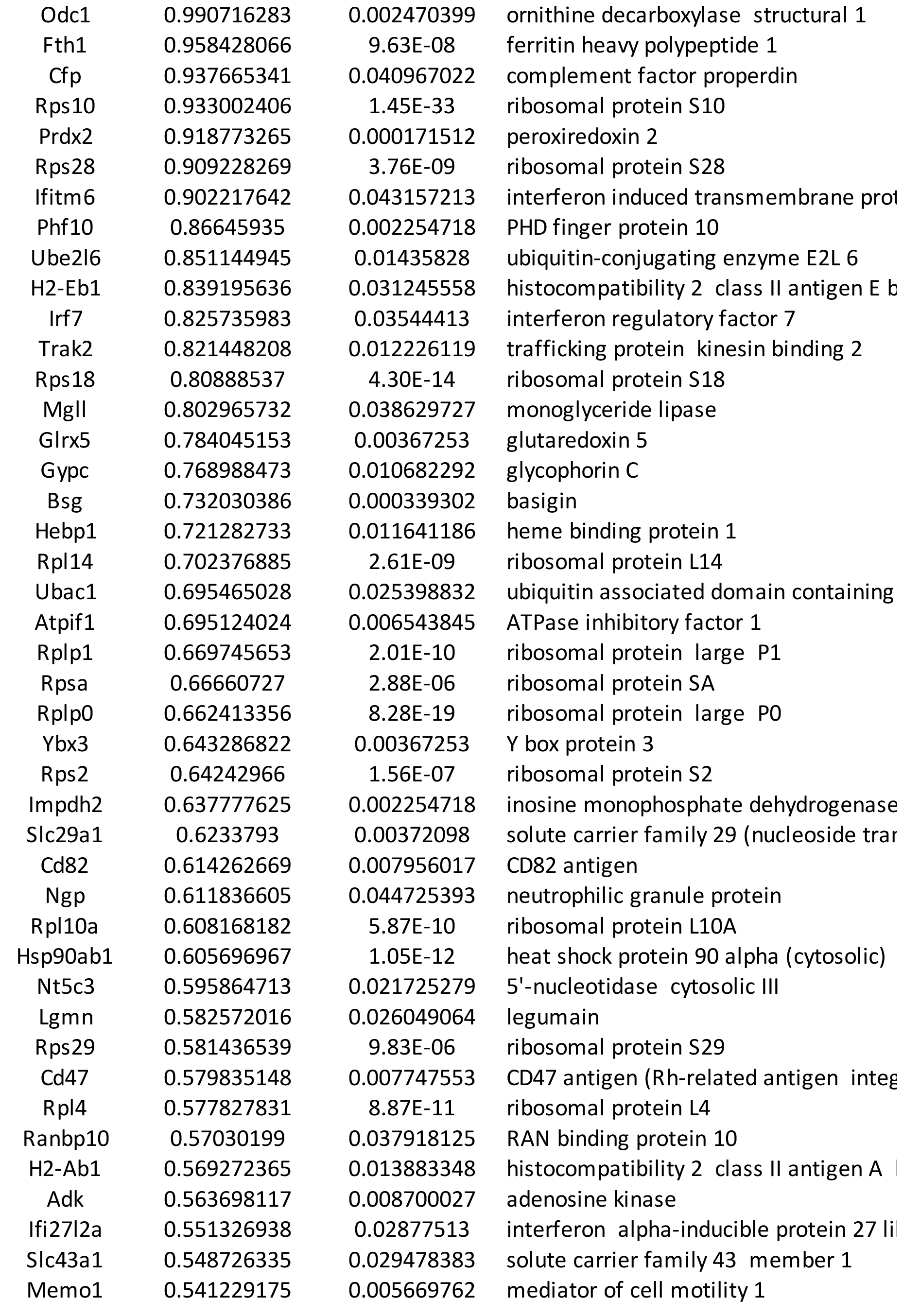


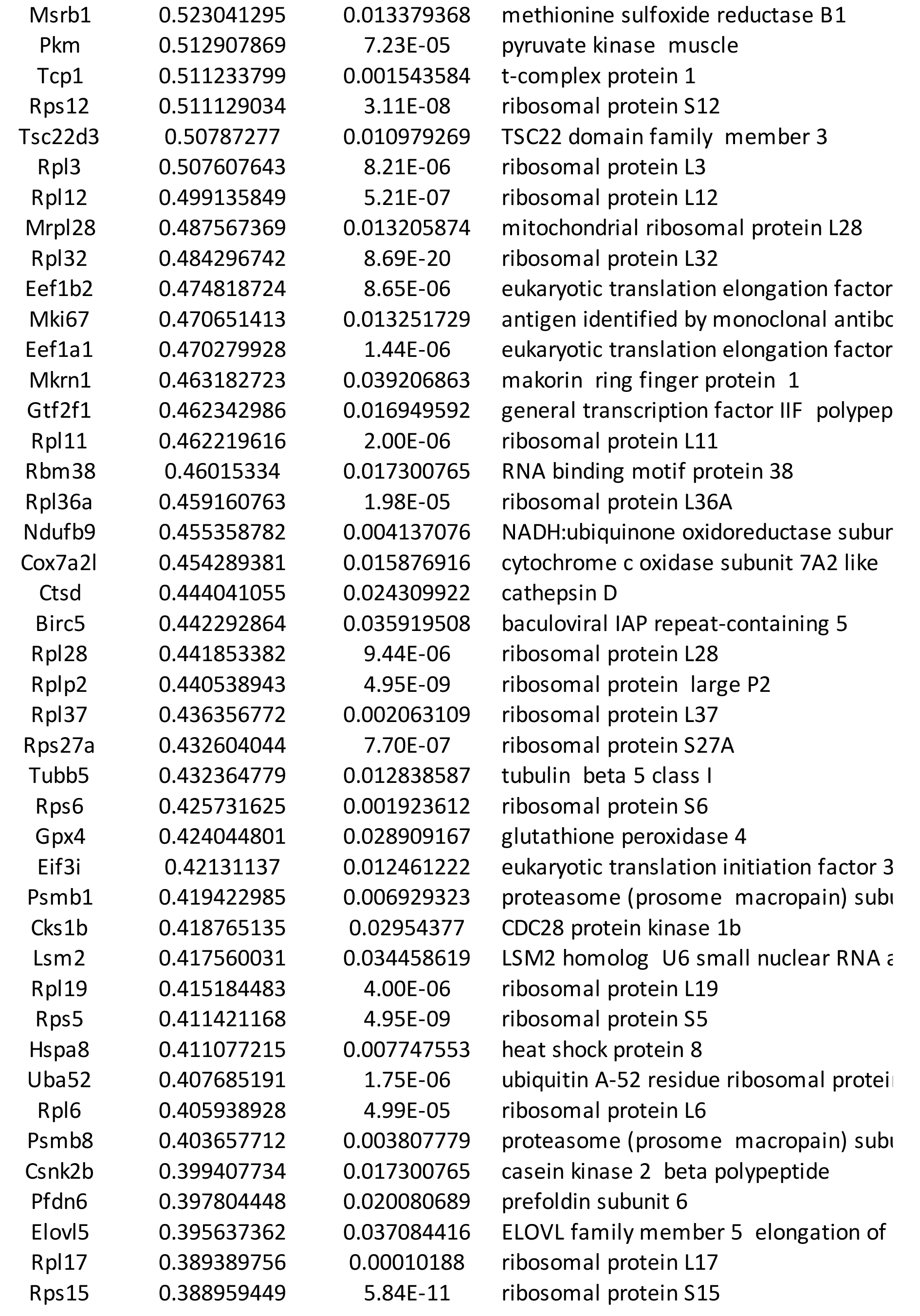


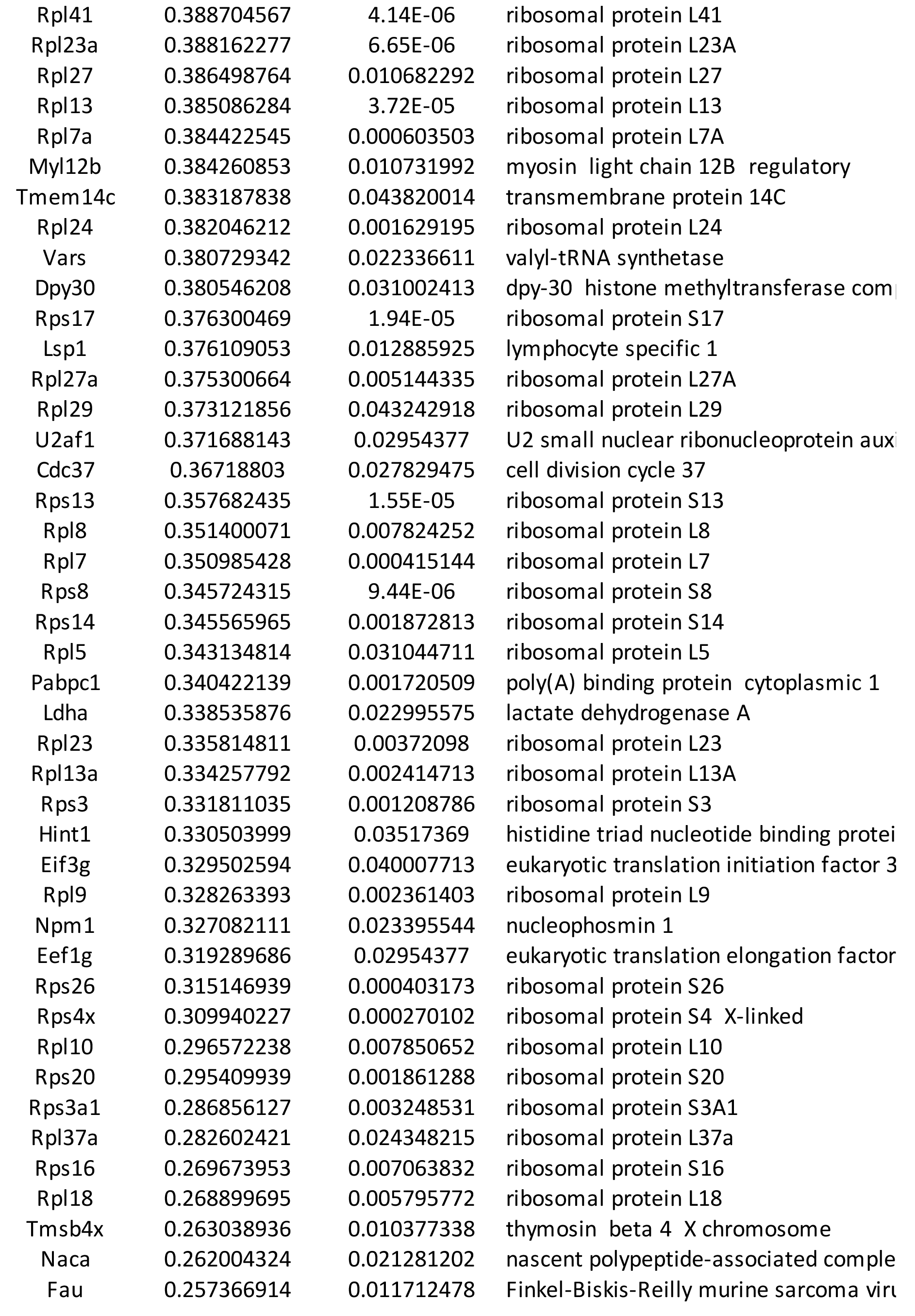


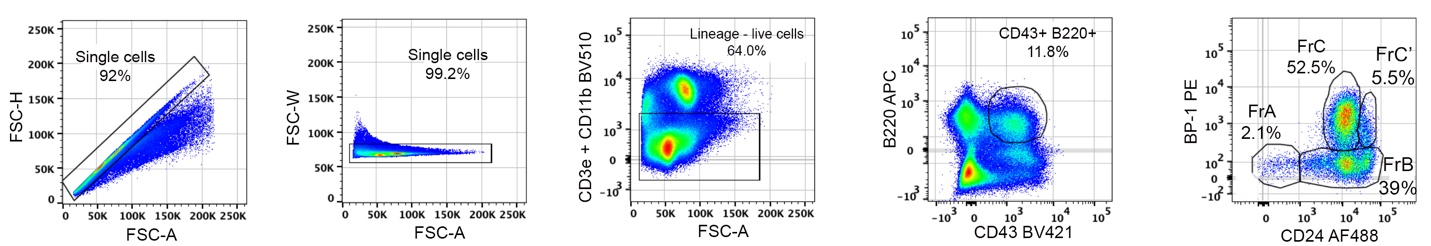


**Supplemental Figure 1:** Preleukemia and Hardy Fraction gating strategy


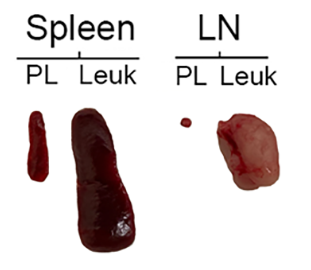


**Supplemental Figure 2**: Leukemic Eμ-Ret mice display enlarged spleen and lymph nodes (LN) compared to age-matched preleukemic (PL) controls.


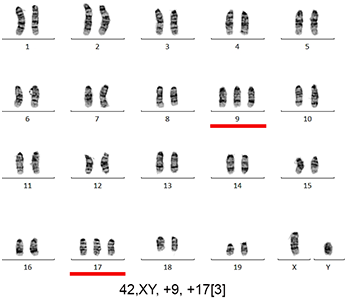
­­­

**Supplemental Figure 3**: Chromosomal trisomy 9 and 17 in primary Eμ-Ret leukemia sample R186-6. (red line indicates trisomy)

**
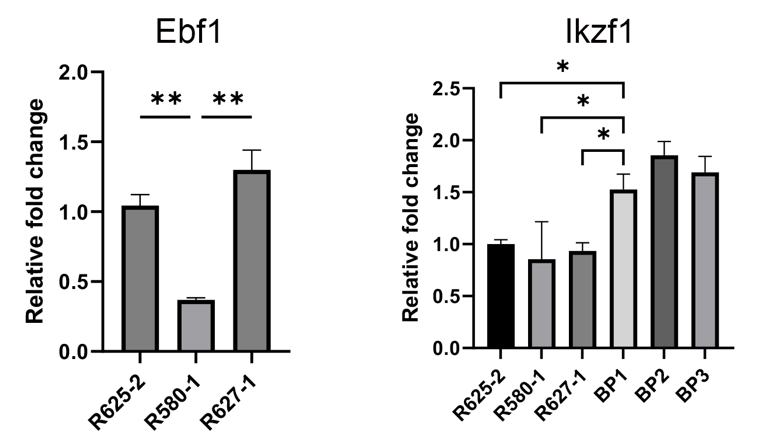
**

**Supplemental Figure 4:** Quantitative PCR analysis of OGM-analyzed leukemia samples. Relative expression of Ebf1 (left panel) and Ikzf1 (right panel) in the 3 leukemia samples and 3 normal BCP cell controls (BP 1-3). (Ordinary One-Way ANOVA followed by post-hoc Fisher’s LSD test, * p<0.05, ** p<0.01. One representative from 3 independent qPCR runs is shown (with a total of 9 technical replicates for each sample). Data normalized to sample R625-2 for both genes. Representative of two independent experiments is shown.


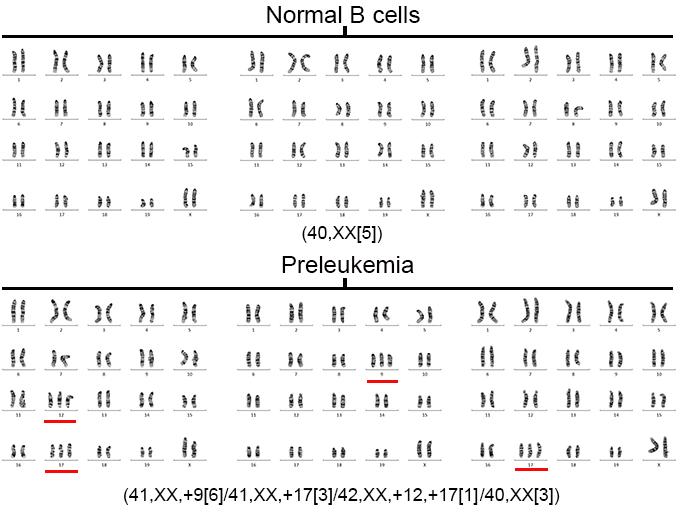


**Supplemental Figure 5: G-Banded karyotyping of cells from preleukemic mouse.** Normal mature B cells (upper panel) and preleukemic BCP cells (lower panel) were purified from the spleen of a preleukemic Eμ-Ret mouse (R224-6) and subject to G-Banding analysis. All mature B cells counted (n=5) showed normal karyotype, while diverse aneuploidy was detected in the preleukemic cell sample. Defined overall karyotypes are shown below each panel.


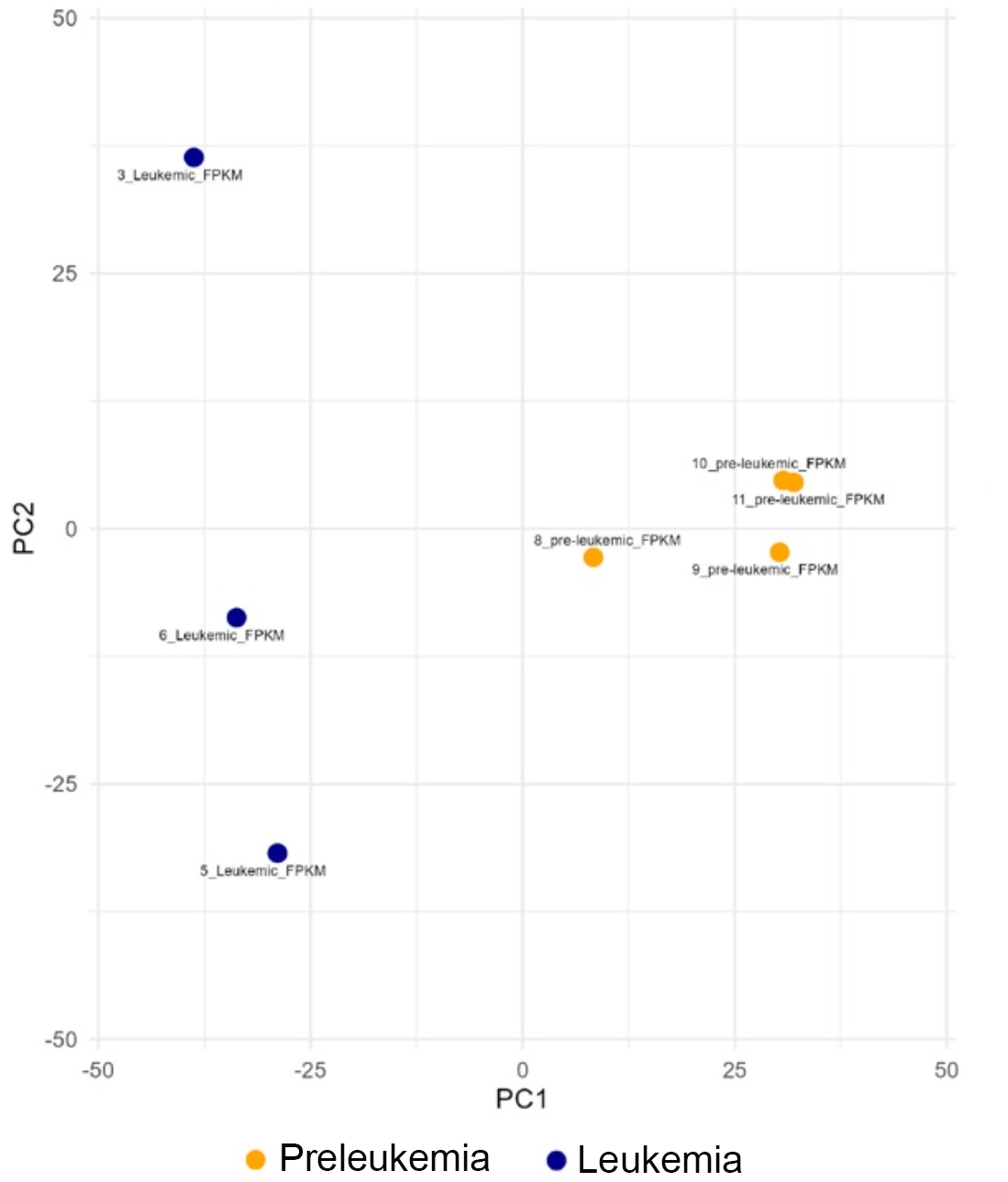


**Supplemental Figure 6:** Principal Component Analysis of RNA-seq data.


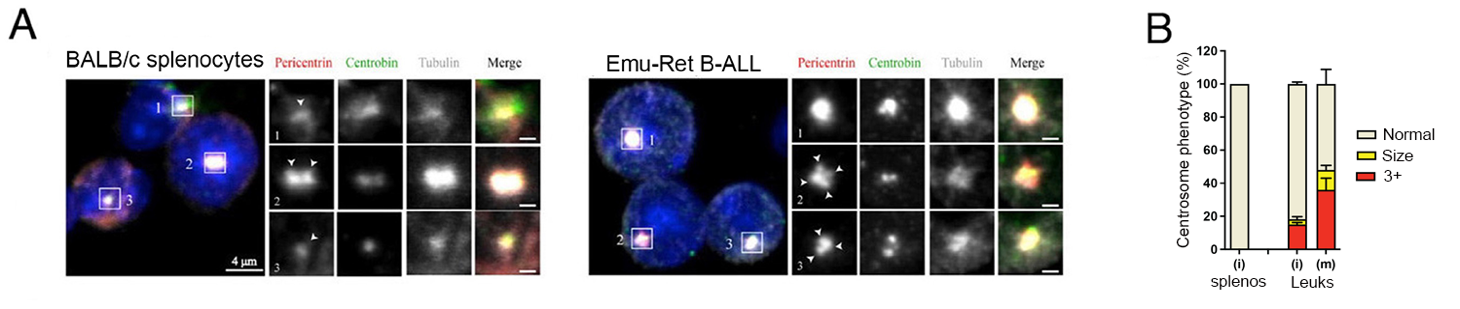


**Supplemental Figure 7: Centrosome amplification in Eμ-Ret leukemia.**

**A**) BALB/c splenocytes (left panel) and aneuploid Eμ-Ret B-ALL cells (right panel) were stained with pericentrin (PCM), centrobin (daughter centriole), β-tubulin (microtubules), and DAPI to enable assessment of centrosomes. All the images are maximum intensity projections of optical sections acquired at 0.5μm intervals through the cell Z-axis. Scale bar = 4μm. The centrosome is highlighted by an arrow in enlarged panel. Scale bar =1μm. **B**) Centrosome abnormalities are detected in interphase and mitotic Eμ-Ret-derived leukemia cells (Leuks) compared to normal splenocyte controls (splenos) from n=2 mice. Centrosome number (red) and size (yellow) were assessed in 20 mitotic (m) and 100 interphase (i) cells. Error bars represent SEM.


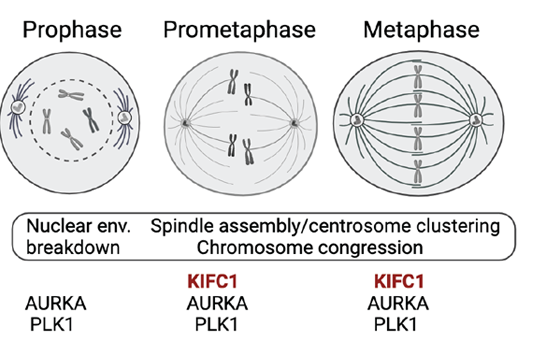
**­­**

**Supplemental Figure 8**: Diagram depicting the mitotic stages of prophase, prometaphase, and metaphase indicating the processes that occur and the selected molecular targets during those stages.

_­­­_
